# Objective homogeneity quantification of a periodic surface using the Gini coefficient

**DOI:** 10.1038/s41598-020-70758-9

**Published:** 2020-09-03

**Authors:** Björn Lechthaler, Christoph Pauly, Frank Mücklich

**Affiliations:** Department of Materials Science & Engineering, Institute for Functional Materials, Saarland University, Campus D3.3, 66123 Saarbrücken, Germany

**Keywords:** Characterization and analytical techniques, Surface patterning, Applied mathematics

## Abstract

The significance of periodic surface structuring methods, such as direct laser interference patterning, is growing steadily. Thus, the ability to objectively and consistently evaluate these surfaces is increasingly important. Standard parameters such as surface roughness or the arithmetic average height are meant to quantify the deviation of a real surface from an ideally flat one. Periodically patterned surfaces, however, are an intentional deviation from that ideal. Therefore, their surface profile has to be separated into a periodic and a non-periodic part. The latter can then be analyzed using the established surface parameters and the periodic nature allows a quantification of structure homogeneity, e.g. based on Gini coefficient. This work presents a new combination of established methods to reliably and objectively evaluate periodic surface quality. For this purpose, the periodicity of a given surface is extracted by Fourier analysis, and its homogeneity with respect to a particular property is determined for the repeating element via a Gini analysis. The proposed method provides an objective and reliable instrument for evaluating the surface quality for the selected attribute regardless of the user. Additionally, this technique can potentially be used to both identify a suitable surface structuring technique and determine the optimal process parameters.

## Introduction

Processes with periodic surface modifications, such as lithography and Direct Laser Interference Patterning (DLIP), are becoming steadily more important. These methods make it possible to design the topography of workpieces over a large area at the micrometer scale and thus change the properties of a system. This results in a wide range of applications.

For example, it is possible to control the wettability^1–4^, to increase the efficiency of solar cells^5–7^, to decrease the contact resistance of electrical connectors^8^, or to improve the tribological^9–12^ and the antibacterial performance^13^.

The periodically modified surface consists of repeating cells, which in the optimal case all have the same property. In reality, there will always be deviations between the individual components due to material and process errors. This means that the surface will never be absolutely homogeneous and imperfections can impair the desired application. Therefore, the ability to objectively evaluate these surfaces becomes critical for process control.

Standard surface values such as roughness or average height are not suitable for this purpose. By definition, they are only sensitive to average variations in the amplitude of the surface heights and not on how the heights are distributed on the surface, which is a critical parameter of periodic surfaces^14^. This is because these parameters were developed to quantify the deviation of a real surface from an ideally flat one. Periodically patterned surfaces, however, are an intentional deviation from the ideally flat surfaces, which renders standard surface parameters alone useless for their description.

Thus, other methods are required to determine the homogeneity of a surface:

One method, for example, is based on the idea that, on a homogeneous surface, it should not matter how this surface is partitioned. The means and variances of a variable should be independent of the size and shape of a segment. The homogeneity is then examined by dividing the area sequentially and classifying the real differences in the distribution of the parameters using a chi-square test^15,16^. The problem is that this can only work on random surfaces without a preferred spatial direction. For periodic surfaces, the calculated variables will always depend on the size and shape of the segments, so that this procedure cannot be easily applied.

Another possibility is to study the spatial variance of the surface parameters. In the work of A.I. Aguilar-Morales et al.^17^ the homogeneity of a laser-patterned with line-like structures is evaluated by analyzing the height fluctuations calculated for arbitrary 1D extractions perpendicular to the lines of the 2D surface profile. This is used to calculate the mean structure height (Rc) and the data dispersion by its standard deviation (SD). Homogeneity is now rated by the ratio between the terms SD and Rc.

This method shows potential for line patterns. With complex periodic patterns, however, it is difficult or even impossible to objectively determine a representative line profile. Depending on user choices, the calculation will yield different results. This applies in particular to surfaces with disturbances.

In addition to mathematical analysis methods, some periodic surfaces also allow an experimental investigation of homogeneity. In the work of Simões et al.^18^ the homogeneity of a periodic line pattern is experimentally investigated. For this purpose, the structured surface is irradiated with white light, and the intensity of the reflected blue light with the wavelength λ is measured at a fixed angle α. The surface behaves like a reflection grating only if the line spacing (g) satisfies the condition:

1 $$\sin \left( \alpha \right) = {\raise0.7ex\hbox{$\lambda $} \!\mathord{\left/ {\vphantom {\lambda g}}\right.\kern-\nulldelimiterspace} \!\lower0.7ex\hbox{$g$}}$$

This leads to constructive interference and makes it possible to measure the intensity of the diffracted light. In case of inhomogeneities, the condition is not perfectly fulfilled, and intensity decreases below the angle α.

The procedure is only applicable for patterns with suitable surface periods. Furthermore, the diffraction pattern of complex surface periods is very complex and not easy to measure. Additionally, the type of inhomogeneities cannot be specified.

All these methods have in common that they can be used to evaluate the homogeneity in principle, but usually only for particular properties and only for simple patterns. In addition, they are often not completely objective and reproducible because of the use of a manually chosen “representative” cross-section.

In order to solve these problems, the approach mentioned by Rossi et al.^19^ should be pursued further. They showed that the homogeneity of alloy microstructures can be evaluated by means of a Gini coefficient, a measure that is usually used in economy. In the outlook, the potential for surface analysis of this technique was demonstrated with a very simple example. A manually selected line profile perpendicular to the lines of a periodic line-like pattern was selected and divided into sections by manually labeling the local maxima and minima based on the known periodicity. Based on this manual segmentation homogeneity of the line profile was calculated by means of Gini coefficient. However, this manual and subjective approach cannot be transferred to general periodic surfaces in the presented way because it relies on the user to recognize the periodicity and label the maxima and minima correctly.

The aim of this research is to find a universal solution for any periodic surface containing regularly recurring elements by developing a modular procedure. To this end, a concept from crystallography is adopted. A crystal can be considered to be composed of a lattice and a motif. The former is a pure mathematical description of the translational symmetry while the latter is the actual structural element that is placed on the lattice points (e.g. an atom, an ion, or even a molecule). The translational symmetry and therefore also the periodicity of the structure can be readily extracted by Fourier transform which yields the reciprocal space representation of the crystal. If we consider a periodic surface structure to be the crystal, a Fourier transform of a height-encoded surface image can be used to quantify its periodicity. The surface can then be decomposed into segments of a size related to the periodicity and attributes of the individual segments can be determined. Finally, the homogeneity of these attributes is calculated with an unequal distribution metric. Note that the obtained periodicity does not depend on the actual shape of the characteristic surface element (e.g. lines, circels, triangles, etc.)

## Methods and materials

The homogeneity will be calculated by using the Gini coefficient as an inequality metric. Therefore, the theoretical background of the Gini analysis is first explained in detail. Subsequently, a description of the experimental procedures follows.

### Theoretical background of the Gini coefficient

The Gini coefficient is an established inequality metric used in the economy to evaluate wealth distributions. In the following, the technique will first be explained in general and then demonstrated where the potential but also the difficulty in the evaluation of periodic surfaces lies.

To develop a suitable surface analysis method, the definition of homogeneity proposed by Rossi et al.^19^ must be reiterated, and its applicability to periodic surfaces determined. The concept of homogeneity has been defined as follows:

"The homogeneity (*H*) of a system is the similarity of its components considering a given attribute *y*."

Referring to the definition, in this work the surface to be examined is considered the system, the repeating surface structures (i.e. the individual segments) are termed the components; and the property whose homogeneity is to be determined is named the attribute.

The Gini coefficient *G* is a measure of inequality distribution that can assume a value between 0 and 1^20,21^. Here, 0 stands for completely equal distribution, and 1 represents completely unequal distribution. In economy, this means that everyone has the same wealth or all the wealth is in the possession of a single person, respectively.

If unequal distribution is equivalent to inhomogeneity, the homogeneity *H* can be simply calculated as:

2 $$H = 1 - G$$

The computed homogeneity is always related to a particular attribute and can only be compared with respect to that particular property. All properties with non-negative values can be used as attributes for this purpose (for example, surface roughness, peak-to-valley (PV) distance, or the distance between individual maxima, but not surface skewness).

For both discrete and continuous distributions, *G* can be calculated using a Lorentz curve, which is a graphical representation of a probability distribution that visualizes relative frequency^21^. Because the main goal of this work is to analyze real measured values, only the discrete case are discussed therein^22^. As described in detail in the previously mentioned paper, the calculation is performed as follows. First, the system is divided into *n* components of equal size. After that, the attribute $y_{i} \ge 0$ is determined for each component *i*. The calculated values are then sorted in ascending order $y_{1} \le y_{2} \le \cdots \le y_{n}$.

Using the average value $\overline{y} = \frac{1}{n}\mathop \sum \limits_{i = 1}^{n} y_{i}$ of the attributes $y_{i}$, the cumulative share of the *i*th attribute can be calculated as:

3 $$L_{i} = \frac{1}{{n\overline{y}}}\mathop \sum \limits_{j = 1}^{i} y_{j}$$

**Figure 1:**
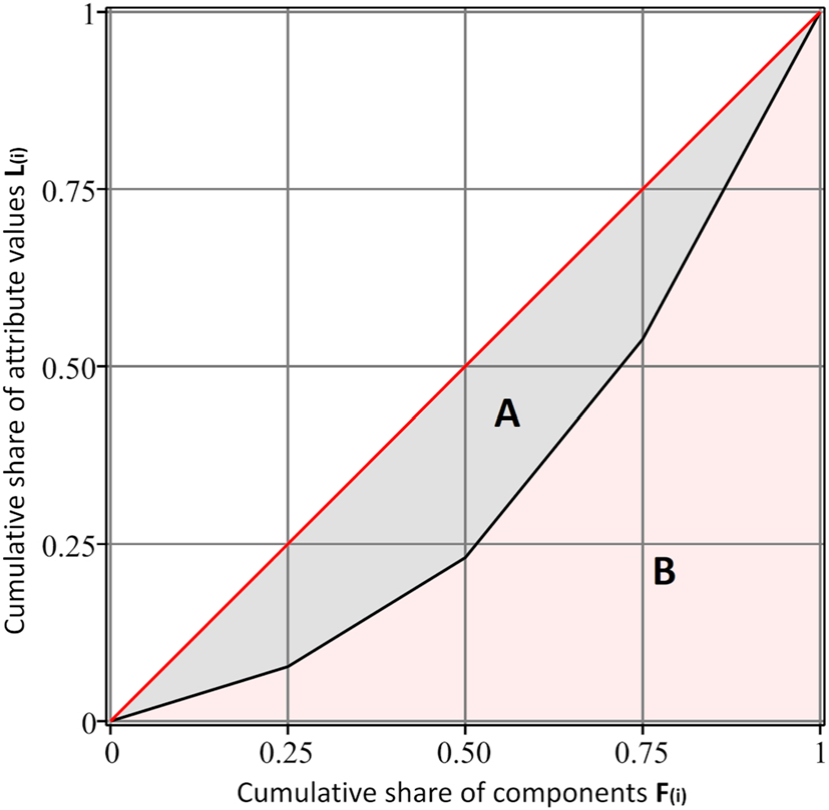
An example of the discrete Lorentz curve. The red line depicts the ideal equal distribution of each attribute with the same value for each component. The black line depicts the real distribution of a system. The Gini coefficient is calculated as the ratio of area A to the sum of areas A and B (A + B).

With $L_{0} = 0$ and $L_{n} = 1$. In the resulting plot of the Lorentz curve (Fig. 1), the ordinate represents the cumulative part $L_{i}$ of the attribute $y_{i}$, while the abscissa denotes the cumulative share *F* of the components calculated using the following formula:

4 $$F_{i} = \frac{i}{n}$$

The red line represents a completely uniform distribution, which corresponds to absolute homogeneity with equal values of all attributes. The black line denotes the real case whose deviation from the perfect homogeneity draws it closer to the abscissa.

The Gini coefficient can then be calculated as the ratio of area A to the total area under the red line A + B:

5 $$G: = \frac{A}{A + B}$$

Because $A + B = \frac{1}{2}$, it follows that:

6 $$G = 1 - 2B$$

The area under the Lorentz curve can be easily calculated numerically:

7 $$B = \frac{1}{2}\mathop \sum \limits_{i = 1}^{n} \left( {F_{i} - F_{i - 1} } \right)\left( {L_{i} + L_{i - 1} } \right)$$

Combining (6) and (2), the homogeneity can be determined as a function of the attribute *y*:

8 $$H_{Y} = \mathop \sum \limits_{i = 1}^{n} \left( {F_{i} - F_{i - 1} } \right)\left( {L_{i} + L_{i - 1} } \right)$$

After inserting (3) and (4) in (8), the following expression for H_y_ is obtained:

9 $$H_{Y} = \frac{2}{{n^{2} \overline{y}}}\left( {\mathop \sum \limits_{i = 1}^{n} \left( {n + 1 - i} \right)y_{i} } \right) - \frac{1}{n}$$

Thus, the homogeneity of a particular attribute can be determined from its values (*y*_*i*_) and their total number (*n*).

An overall homogeneity *H* can be defined for different attributes z, y, x… etc. of the same system, as follows^19^:

10 $$H_{total} = H\left( z \right) \cdot H\left( y \right) \cdot H\left( x \right) \cdot \ldots$$

It should be noted that from a physical point of view, it is difficult to interpret the obtained results when the number of attributes is high. In this case, it is more reasonable to directly compare individual attributes. To ensure comparability in specifying the homogeneity of a system, it must always be stated with respect to which attributes the parameter is defined.

**Figure 2:**
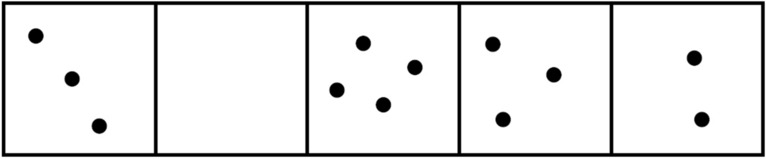
In this example, the entire area (the system) is divided into 5 subsections (components (*n*)), which contain different numbers of dots (attribute (*y*)).

To illustrate this principle, the following example is considered. Figure 2 shows five boxes filled with different numbers of dots. The five boxes represent the five components of the system. The number of dots corresponds to the attribute of the specific component. The homogeneity of the filled boxes is calculated as follows. In the first step, prerequisite conditions are verified. The number of dots that fill a box represent an attribute with $y_{i} \ge$ 0, and sorting the filled sections by size yields $y_{1} = 0, y_{2} = 2, y_{3} = 3, y_{4} = 3, \;and\;y_{5} = 4$. Using Eq. (5), the homogeneity value of 0.7 is obtained. For the distribution with $y^{\prime}_{1} = 2, y^{\prime}_{2} = 3, y^{\prime}_{3} = 3, y^{\prime}_{4} = 3, and y^{\prime}_{5} = 4$, a value of 0.83 is calculated, which corresponds to a greater degree of homogeneity.

The Gini analysis was performed as described with a self-written code in Maple (Maplesoft).

### Experimental implementation

**Figure 3:**
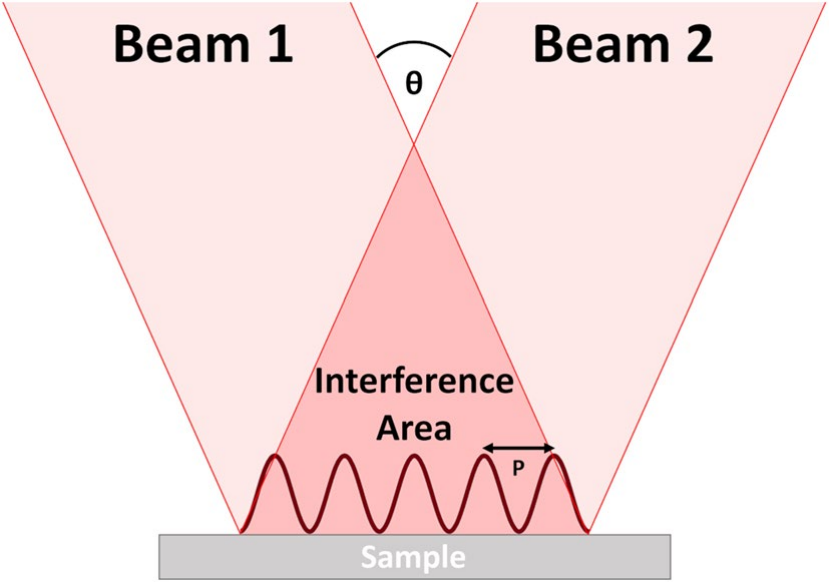
Two overlapping coherent laser beams that produce an intensity distribution, $\sim sin^{2}$, which periodically melts the surface and creates a line-like pattern.

Samples with periodic surfaces are created using the Direct Laser Interference Patterning (DLIP) process. A coherent (ultra-)short pulsed laser beam is split and overlaid under a certain angle between 0° and 180° to form an interference pattern on the sample surface. Depending on the number of beams and angles, different periodic intensity distributions can be generated. In the simplest case, a periodic line-like pattern is obtained from two-beam interference if an appropriate laser pulse energy density is chosen (Fig. 3). The distance between the maxima, i.e. the periodicity P of the surface, can be calculated using Eq. (11). Under practical conditions (angle, wavelength, and type of laser system), periods between 0.5 and 30 µm can be achieved. A general overview of different methods to realize the procedure can be found here^23^.

11 $$P = \frac{\lambda }{{2 \cdot \sin \frac{\theta }{2}}}$$

The first set of samples was structured using a nanosecond laser (Quanta Ray PRO 290, Spectra Physics; repetition rate: 10 Hz, pulse duration: 10 ns, wavelength 532 nm). Owing to the resulting interference pattern, high temperature gradients were formed between the minima and maxima, leading to Marangoni convection, which creates a line-like topography^24^. The energy maxima correspond to the topography minima on the surface and vice versa (details of the utilized method can be found in the study by Mücklich et al.^25^). In the present work, the described procedure was performed on a stainless steel sample (X5CrNi18-10, Brio Kontrollspiegel GmbH). The structural period as distance between the maxima was 8.8 µm. In order to pattern a surface completely, single shot spots of 1.1 × 1.0 mm^2^ size are stitched together. This results in double and quadruple exposures at edges and corners of individual spots, respectively. As the number of exposures increases, the pattern quality degrades due to multiple melting events. A detailed description of the type of defects that occur can be found here^26^.

The second set of samples was structured using a picosecond laser (PX200-3-GH, ps-INNOSLAB-Laser, EdgeWave; repetition rate: 10 kHz, pulse duration: 10 ps, wavelength: 1,064 nm). The short pulse duration shifts the laser-matter-interaction away from melting and towards ablation, even under multiple exposure conditions. Further details regarding this procedure can be found in the publication of Bieda et al.^27^. This method was applied to tungsten discs (purity > 99.95 wt.-%, Plansee) with a diameter of 20 mm and a thickness of 5 mm, which were cut off a rod and polished. The laser structure period was 8.8 µm.

For further analysis, grayscale height profile images of the surfaces were recorded using a confocal laser-scanning microscope (LSM) (OLS4100, Olympus). A 50 × objective lens with the numerical aperture NA = 0.95 was used with an optical zoom of 1.1 × for each observation. In each case, a data field of 1,024 × 1,024 pixels was recorded with a pixel size of about 0.23 × 0.23 µm^2^, which corresponds to a field of view of about 235 × 235 µm^2^.

To extract the period, a discrete Fourier transformation was performed on the obtained height profiles using the free software “Gwyddion”^28^. It should be noted that the software only allows pixel-precise filtering of the data for the inner part of the Fourier images. As a result, the frequency analysis was focused on this area. In the examples shown, this has no noticeable influence on the calculated period.

The subsequent calculation of the period and the drawing of the colormap was carried out with Origin (OriginLabs).

## Developing a method for homogeneity analysis

**Figure 4:**
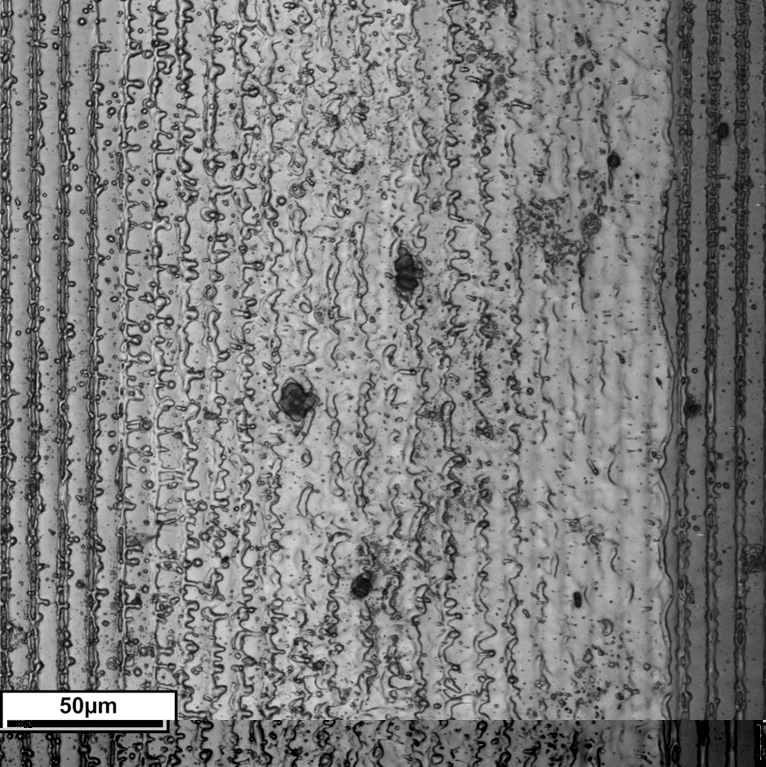
An intensity image of an inhomogeneous laser-textured steel surface obtained with a confocal laser-scanning microscope. The underlying periodic structure is clearly visible.

The main obstacle to calculate the Gini coefficient for surfaces is the determination of the components. If the previously described procedure is applied to a periodically structured surface (Fig. 4), the challenge of classifying its components objectively and consistently must be addressed. An arbitrary grid (utilized in the previous example as well as in the analysis of microstructures by Rossi^19^) is not applicable here because it would produce different results for different positions. It is clear that the periodic structure needs to be accounted for when choosing the components.

In the following, it is explained how the type, position, and size of the periodic pattern can be determined objectively and reproducibly using Fourier analysis, and how the components are specified by this. Furthermore, the conditions that must be fulfilled for the attributes to be evaluated by Gini analysis are also defined.

### Specification of surface components

To define the proper segments (components) of the surface that account for its periodicity, Fourier analysis is be performed. For this purpose, the height profiles were first determined using a confocal laser-scanning microscope. The information obtained in this way was converted into grayscale images, in which the brightest area is represented as the highest one, and the darkest region as the lowest one.

Because the image of a surface is resolved pixel by pixel, discrete Fourier analysis is conducted by converting the image pixels into a matrix. The position of the pixel of line x and column y corresponds to the matrix entry [*x, y*] with a specific gray value. A two-dimensional (2D) Fourier transformation converts the image into an object in the Fourier or so-called frequency space. The brighter a point, the higher its intensity, and the more often this spatial period (distance) occurs in the real space. Note that the general result of the Fourier transform not depend on the actual scale of the original image. For the sake of representation, inverted images are used in this work.

**Figure 5:**
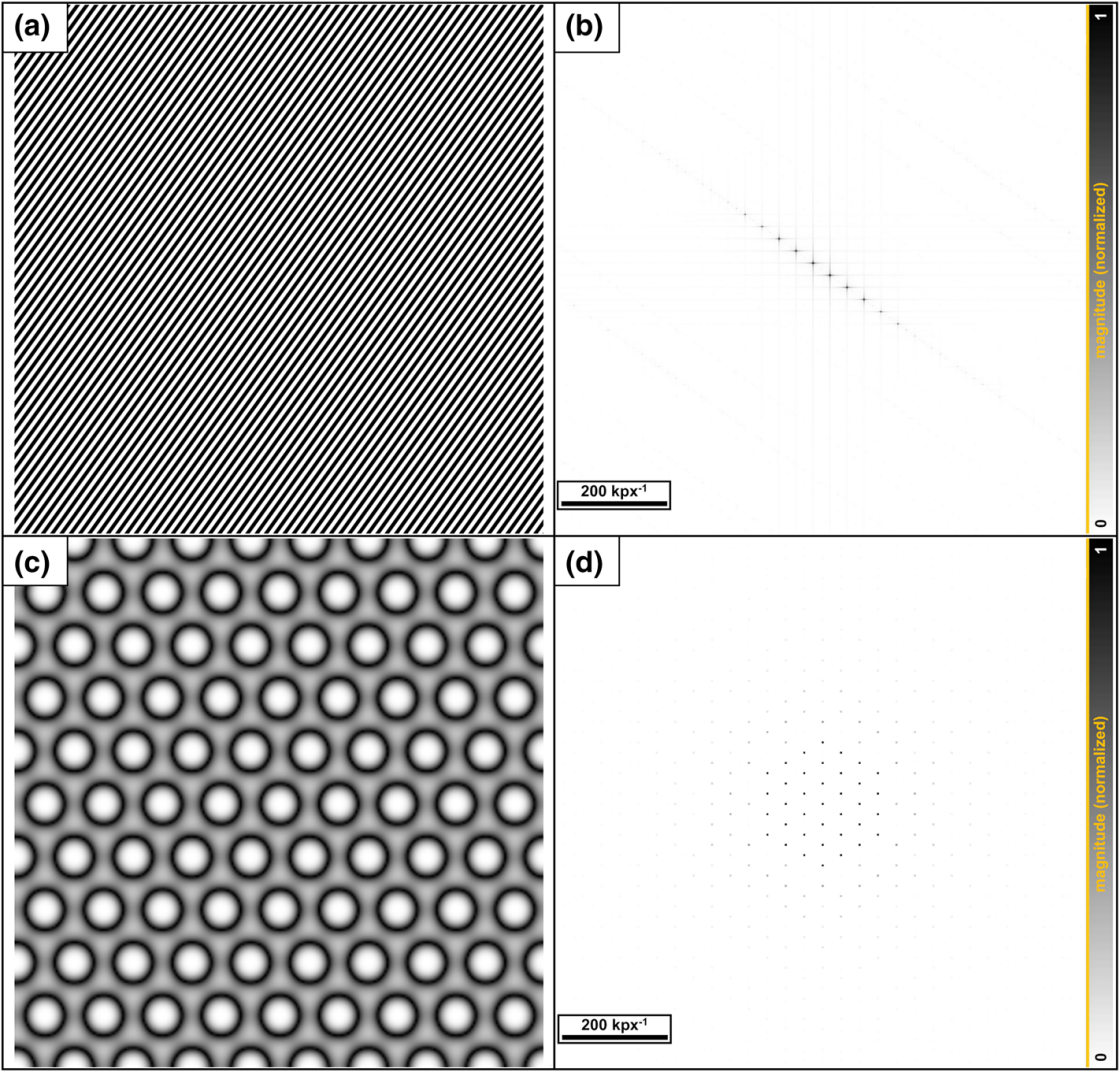
(**a**) An oblique linear profile with a resolution of 3,543 × 3,543 pixel and (**b**) the corresponding (inverted) Fourier transform (**c**). A simulated perfect hexagonal surface with (**d**) the corresponding (inverted) pattern in the Fourier space. Here the original image was reduced to 256 × 256 pixels (px) before the transformation in order to increase the visibility of the main frequencies.

In addition to the main frequency, higher harmonics with lower intensities can also be observed. Examples using artificially generated images are shown in Fig. 5. The diagonally aligned lines in the local space (Fig. 5a) lead to a distribution in the Fourier space arranged at the same angle (Fig. 5b). Figure 5c below shows a simulated hexagonal structure with a corresponding image in the Fourier space (Fig. 5d). The single maxima often have cross-shaped extensions owing to the rectangular shapes of the pixels (px), which generate additional frequencies.

**Figure 6:**
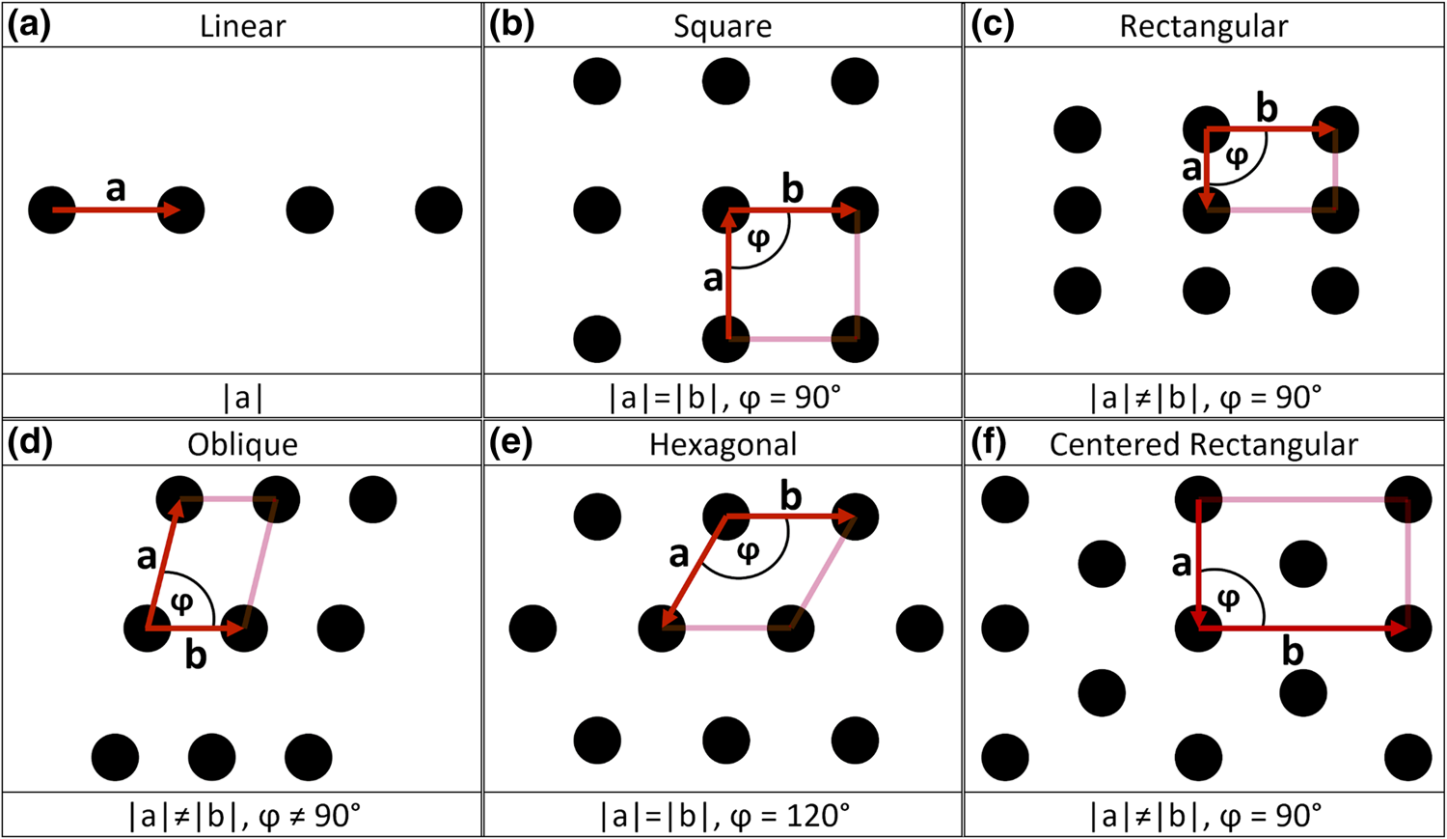
One 1D and five 2D Bravais lattices. Five of these structures are primitive (linear (**a**), square (**b**), rectangular (**c**), oblique (**d**), and hexagonal (**e**)). The only non-primitive structure corresponds to the centered rectangular lattice (**f**).

According to the Bravais classification^29^, periodic surfaces have repetitive components that can be arranged only in six different patterns (Fig. 6). This includes one 1-dimensional (Fig. 6a) and five 2-dimensional lattices (Fig. 6b-f).

#### Division procedure example: 1D case

**Figure 7:**
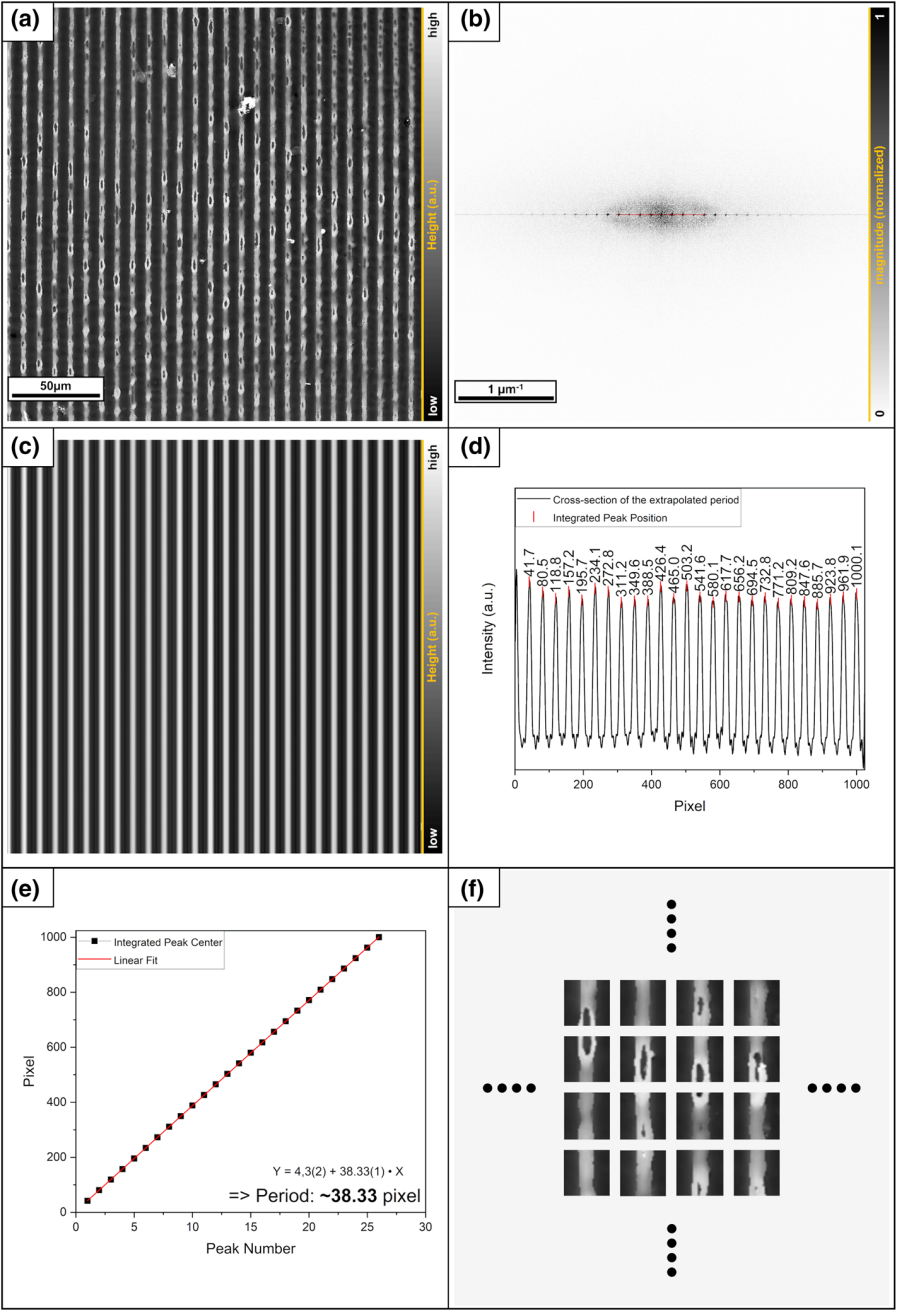
(**a**) Original LSM surface image, (**b**) Fourier transformed, everything except the red part will be filtered out (**c**) reverse transform of the part marked red. (**d**) Cross section of the reverse transformed imaged including the integrated peak positions. (**e**) Calculation of the distances between the peaks. (**f**) An excerpt of the decomposition of the original data with the evaluated results.

Surface components are represented by the grayscale height-profile image of a laser-textured sample in Fig. 7a. First, a Fourier transform is performed on this image (Fig. 7b). The main frequencies are arranged horizontally. From this it can be deduced that this is a 1-dimensional case (like Fig. 6a) and that the repeating components line up without slope. The next step is the elimination of all frequencies in the Fourier space, except for the domain of the main frequencies including the first few harmonic (denoted by the red points). It should be noted that more harmonics could be included in the calculation. As described in “Experimental implementation”, the reduction is due to limitations of the software used, but this has no negative effect on the result in the examples shown.

Because information is contained in one dimension in the linear case, the data can be reduced to a line with a 1-pixel height mask. The filtered image is then transformed back into the real space, which displays only the periodic part of the structure (Fig. 7c). Since all disturbing elements have been removed, an objective classification can be carried out on this reconstructed surface. For this purpose, the position of the peaks is first determined using the integral center of gravity (Fig. 7d). The distance between the peaks |a| is calculated by a simple linear fit of the peak positions (Fig. 7e). This defines the width of the unit cell. In the 1D case, the height of the unit cell is limited only by the image section. If the cells are tilted as shown in Fig. 5a, the individual cells have different heights. To avoid this situation, it is suggested that a cell with a height equal to its width should be selected (this is not mandatory and could also be defined differently.).

In principle, the center of the cell can be selected arbitrarily. The surface is always evenly covered by stringing them together. In this study, either the topography maximum or the topography minimum is selected as the center of the unit cell. Finally, the indices of these resulting cells can then be transferred to the original surface data matrix (Fig. 7f) and serve as components for the Gini analysis.

It should be noted that data recording is discrete, but the real period is continuous. So the decomposition will usually not be absolutely perfect. It is therefore recommended to adjust the positioning of the center of the cell to the attribute in order to minimize possible errors.

This approach advantageously allows a division procedure that is unambiguous and independent of the operator, which solves the problem of objective component determination. The described process can be applied to any periodic surface. Similar to the Bravais lattice, unit cells can be produced for each sample to form a basis for the objective and reproducible Gini analysis.

#### Division procedure example: 2D case

**Figure 8:**
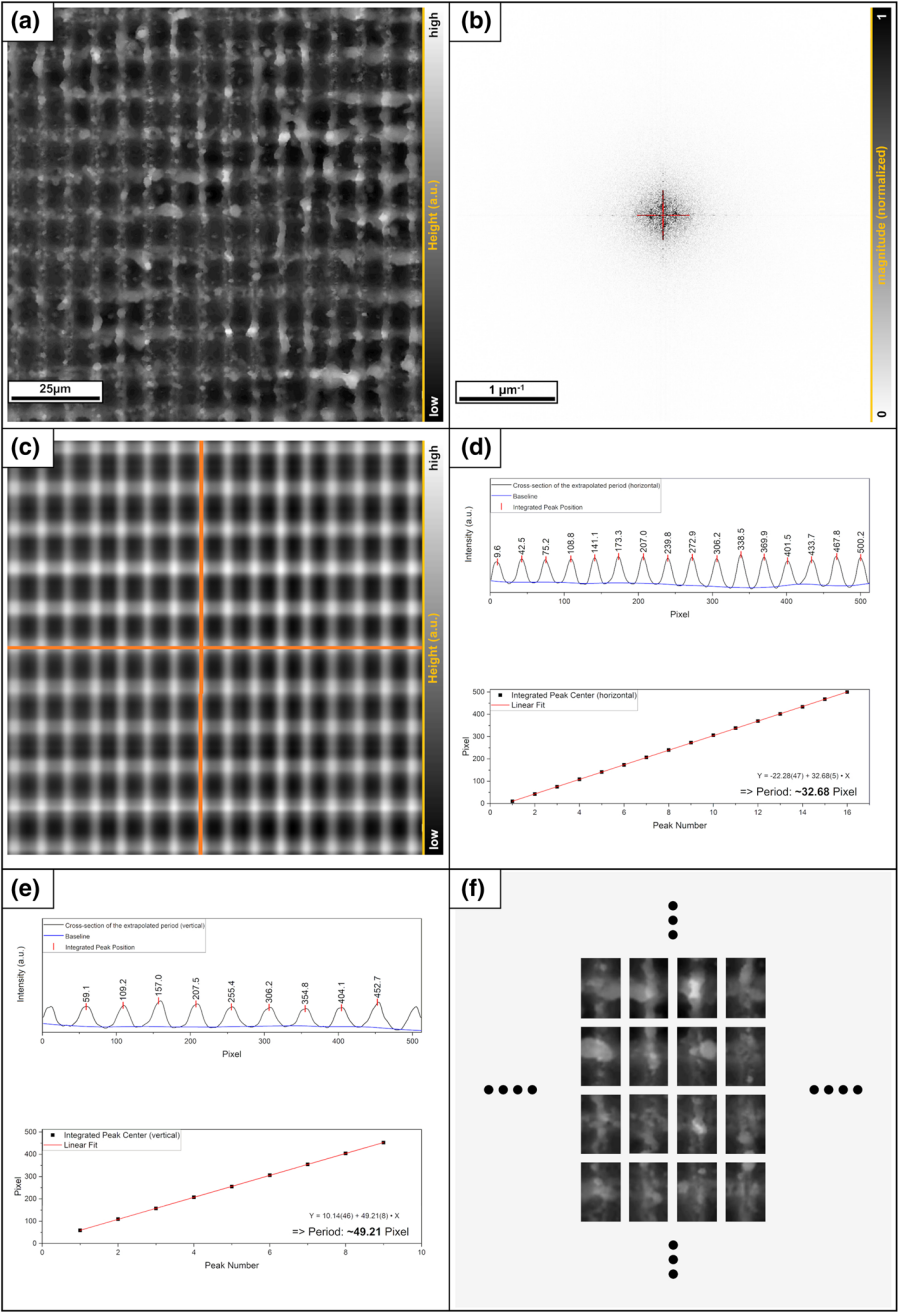
(**a**) Original grayscale image of a cross pattern (512 × 512 pixel), (**b**) the Fourier transformed image, the red part contains the main frequencies. (**c**) Shows the reverse transformed, the orange lines (symmetry axes) mark the positions of the cross-sections for the calculation of the cell size. (**d**) and (**e**) The determination of the distance between the components in the respective symmetry axis. (**f**) An excerpt of the decomposition of the original data with the evaluated results.

In the following, a subdivision for the 2D case is shown. For this example, the sample surface was artificially created by first digitally enlarging the data of a line pattern, then rotating it by 90° and finally overlaying it with the original data set. This was done by simple addition and averaging of the data per pixel. The result was then plotted as a grayscale height profile (Fig. 8). It is important to note that this and much more complex periodic patterns can be realized with a suitable interference setup for real applications^30–32^.

The procedure is nearly the same as in the 1D case. First, a Fourier transform is performed on this image. This time the main frequencies are arranged perpendicular to each other and spread over 2 dimensions. For this reason, the main frequencies, including the first few harmonics, have to be filtered on these two axes. The filtered image is then transformed back into the real space, which displays only the periodic part of the structure. The extraction of the period is now done in the directions given by the main frequencies (direction of a and b, Fig. 6c). A cell now has the respective period as height and width and the peak center is located in the middle. In the 2D case, the complete cell is specified so that there are no degrees of freedom as in the 1D case. The indices of these resulting cells can now be transferred to the original surface data matrix and serve as components for the Gini analysis.

Alternatively, the period of the corresponding direction can also be read out by applying a cross-section to the frequency spectrum and determining the dominant frequency by a fit.

Due to the limited resolution of the images, the value determined in this way often has too large a deviation from the real period. It is therefore advisable to use the overlay of a wider frequency range as described above.

### Attribute determination

Once the surface components are defined, the attributes must be calculated in the subsequent step (the resulting homogeneity will be applicable to only these attributes). The selected property must not have negative values and must be sortable^14^.

**Figure 9:**
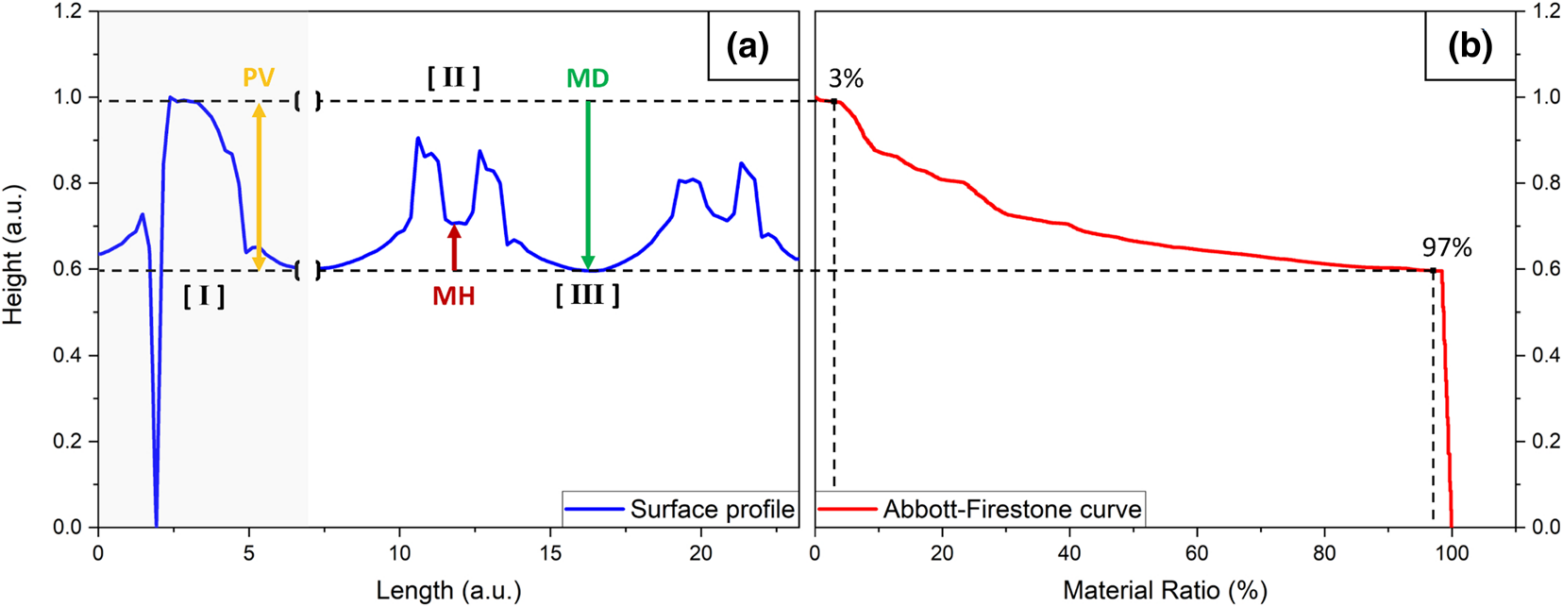
In (**a**) the cross section for a real profile can be seen in blue. [I] marks a closed peak with a measurement artifact, [II] a disturbed maximum with an open peak and [III] a smooth melted area between two peaks. In (**b**) the corresponding Abbott–Firestone curve is shown in red. To eliminate spikes, the 3% peak material volume and 3% valley void volume are removed. This is performed globally for the entire sample area for the Mid-Height (MH) and the Mid-Depth (MD) attributes and per component for the peak-valley distance (PV). Note that this is a 2D illustration to explain the principle, but the parameters were calculated in 3D.

In the following sections, periodic laser structures will be investigated, and the surface data will be obtained via a laser scanning microscope. During recording, measurement artifacts can occur in the form of negative or positive spikes. These can falsify the calculation of the attributes severely and must be eliminated first. For this purpose, 3% peak material volume and 3% valley void volume were removed from the surface data. This is achieved using the Abbot–Firestone curve^33^. The disturbance spikes have large amplitudes but only a very small volume and are eliminated in this manner. The principle is shown in Fig. 9 for a better understanding on a 2D cross-section. For this work, these values were calculated for the volume of the component/sample:

The following attributes were selected for the homogeneity analysis (see Fig. 9):

1. Peak-Valley distance (PV):
  For this attribute, the center of the unit cell is placed on the topography maxima of the period. Then, 3% peak material volume and 3% valley void volume were calculated and removed separately for each component. Finally, the distance from the plane passing through the highest point to that passing through the lowest point is calculated.
2. Mid-Height (MH):
  Here, the center of the unit cell is placed again on the topography maxima of the period. Then, the material ratio is calculated for the entire area, and the lowest 3% is removed. The remaining lowest point is defined as the global minimum.
  The mid-height is now calculated as the height of each cell center with respect to the global minimum. The purpose is to distinguish between open and closed topography maxima (see Fig. 7f). It is clear that this value will not detect every hole, but only works approximatively.
3. Mid-Depth (MD):
  Here, the center of the unit cell is placed on the topography minimum of the period. Then, the material coat is calculated for the entire area and the top 3% is deducted. The remaining highest point is defined as the global maximum.
  The Mid-Depth value is now calculated as the distance of each cell center to the global maximum.

Regardless of the chosen attribute, it is imperative to note under which conditions the parameters are recorded. The homogeneity of different systems can only be compared if the values have been determined under equivalent conditions.

In addition, it is useful but not necessary to display the attributes as a location-dependent heatmap. In this manner, the exact locations of the deviations can be visualized, and it can be easier to detect the origin of disturbances.

## Results and discussion

In the following sections, the previously developed procedure is tested on real surfaces. For this purpose, two materials were interference-patterned by different methods and subsequently examined to determine their homogeneities with respect to three different attributes.

### Surface decomposition and homogeneity analysis

**Figure 10:**
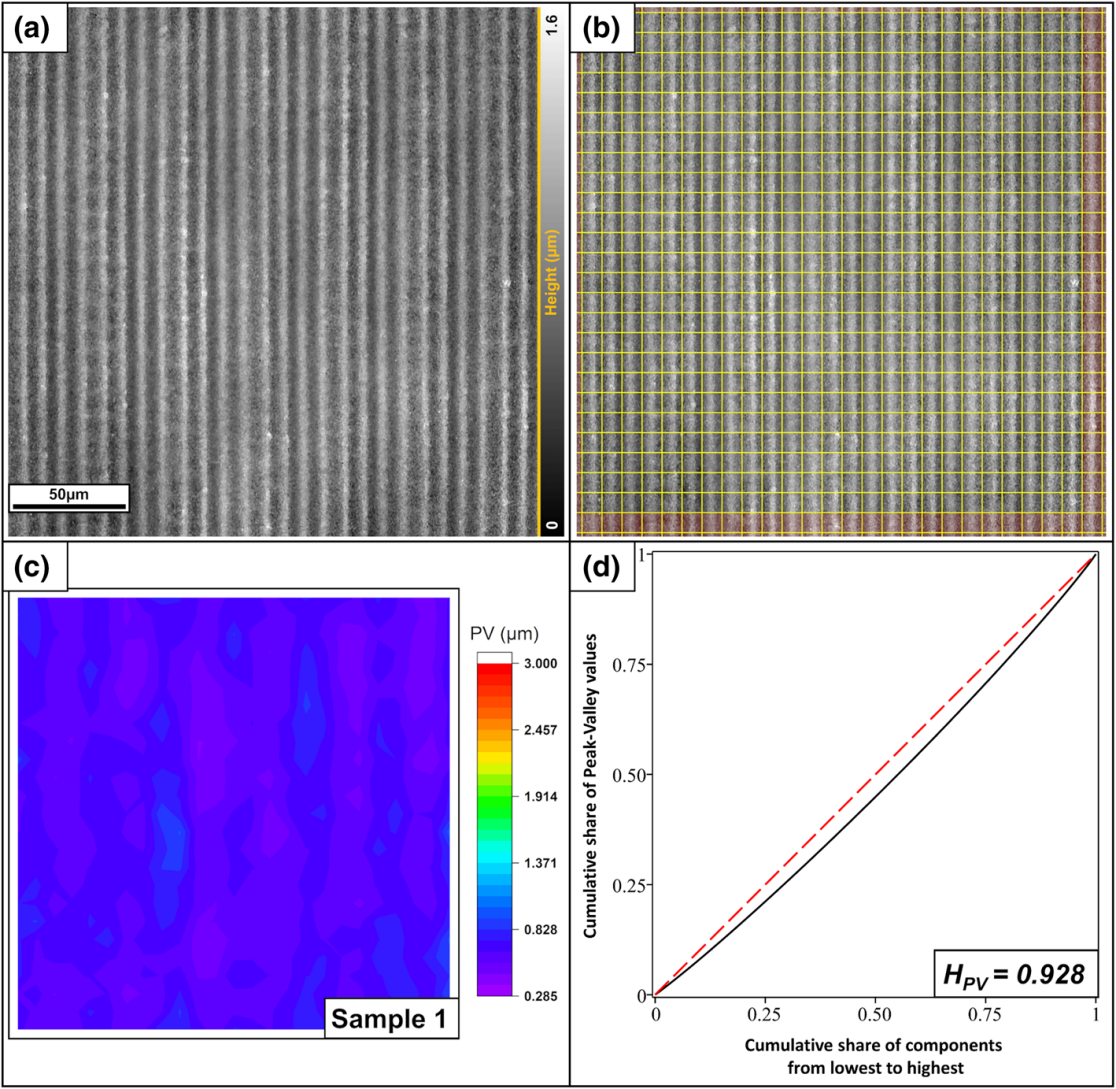
(**a**) LSM image of a tungsten sample patterned with a picosecond laser. (**b**) Splitting into the individual components. The red area was not used to calculate the homogeneity. (**c**) Color map of the PV-values. (**d**) Gini curve of the PV distances, yielding a homogeneity of H_PV_ = 0.928.

The first sample (Fig. 10) is tungsten processed with the picosecond laser. The period is approximately 8.8 µm, which yields a corresponding square decomposition of the surface. The first 25 × 25 cells were used for the evaluation. The color map reveals that the surface of the sample exhibits a relatively high homogeneity (H_PV_ = 0.928), but it indicates that a second period is hidden under the main one. This effect results from the manufacturing process. When structuring the sample, the surface is scanned in lanes of ~ 55 µm width. For a homogeneous result, these tracks are partially overlapped. This implies that some areas of the surface are irradiated more than others, leading to slightly stronger ablation. In the studied case, this effect is very small (approximately 0.08 µm height difference), so it has a very small effect on the PV distance.

**Figure 11:**
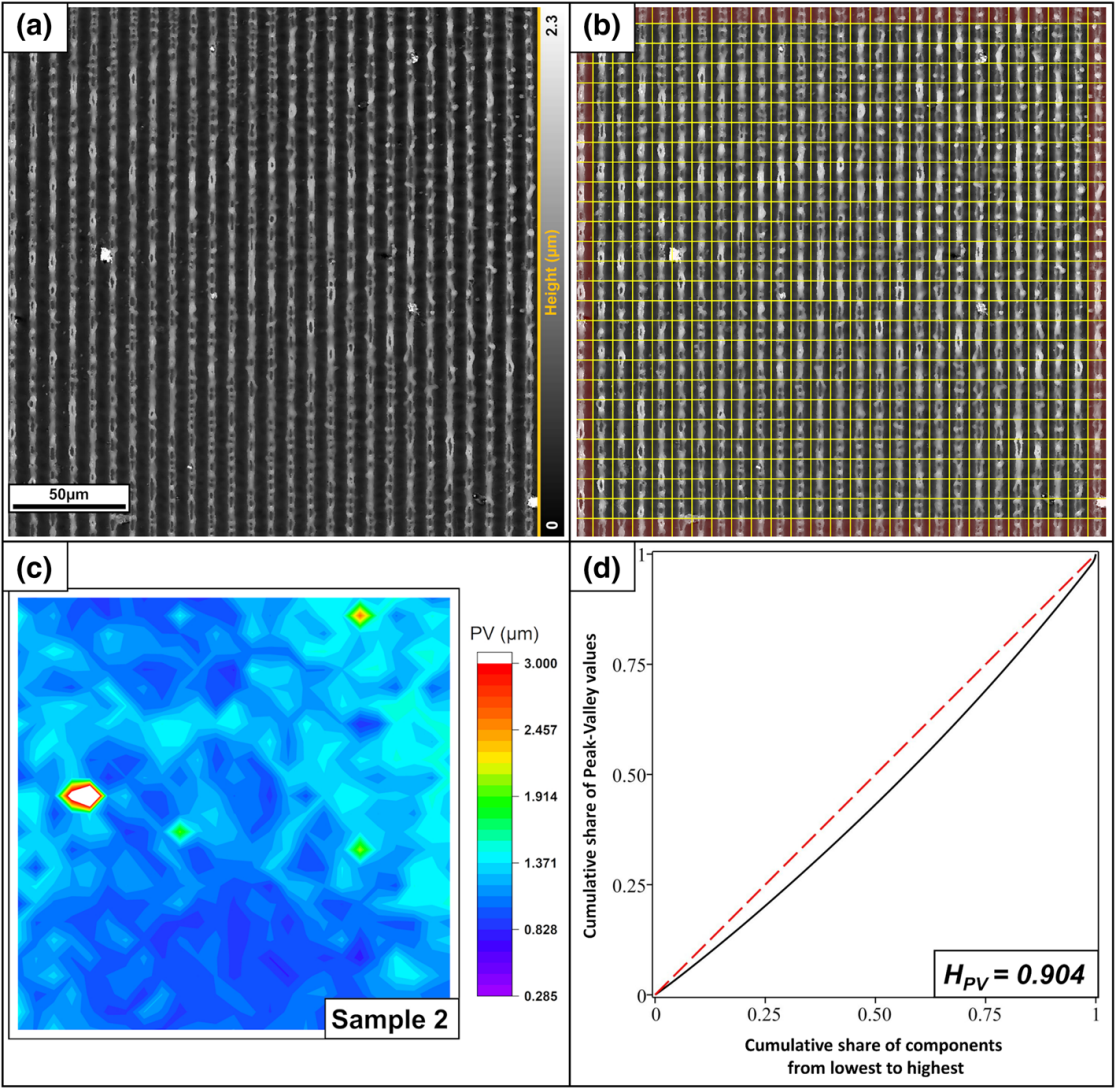
(**a**) LSM grayscale height profile image of a polished steel sample patterned with a nanosecond laser. (**b**) Splitting into the individual components. The red area was not used to calculate the homogeneity. (**c**) Color map of the PV values. The four green/red peaks are caused by melting drops. (**d**) Gini curve of the PV distances, yielding a homogeneity of H_PV_ = 0.904.

The structure of sample 2 was created by applying a two-beam nanosecond-laser interference to a polished steel surface (Fig. 11). The image shows the center of a single square interference spot of approximately 1 mm^2^. Diffraction effects and other disturbances occur primarily at the edges of the spot; therefore, the displayed area is quite homogeneous. The structure period was 8.8 µm. The middle 25 × 25 cells were used for the analysis. The color map displays four extreme values depicted in green and red. A comparison with the original pictures shows melt droplets at these positions. Otherwise, the attribute values are almost identical for different cells, as indicated by the high homogeneity. Note that the chosen attribute (PV distance) is not affected by the cavities in the topographic maxima (see the magnification in Fig. 7f). This finally yields a homogeneity value of H_PV _= 0.904.

**Figure 12:**
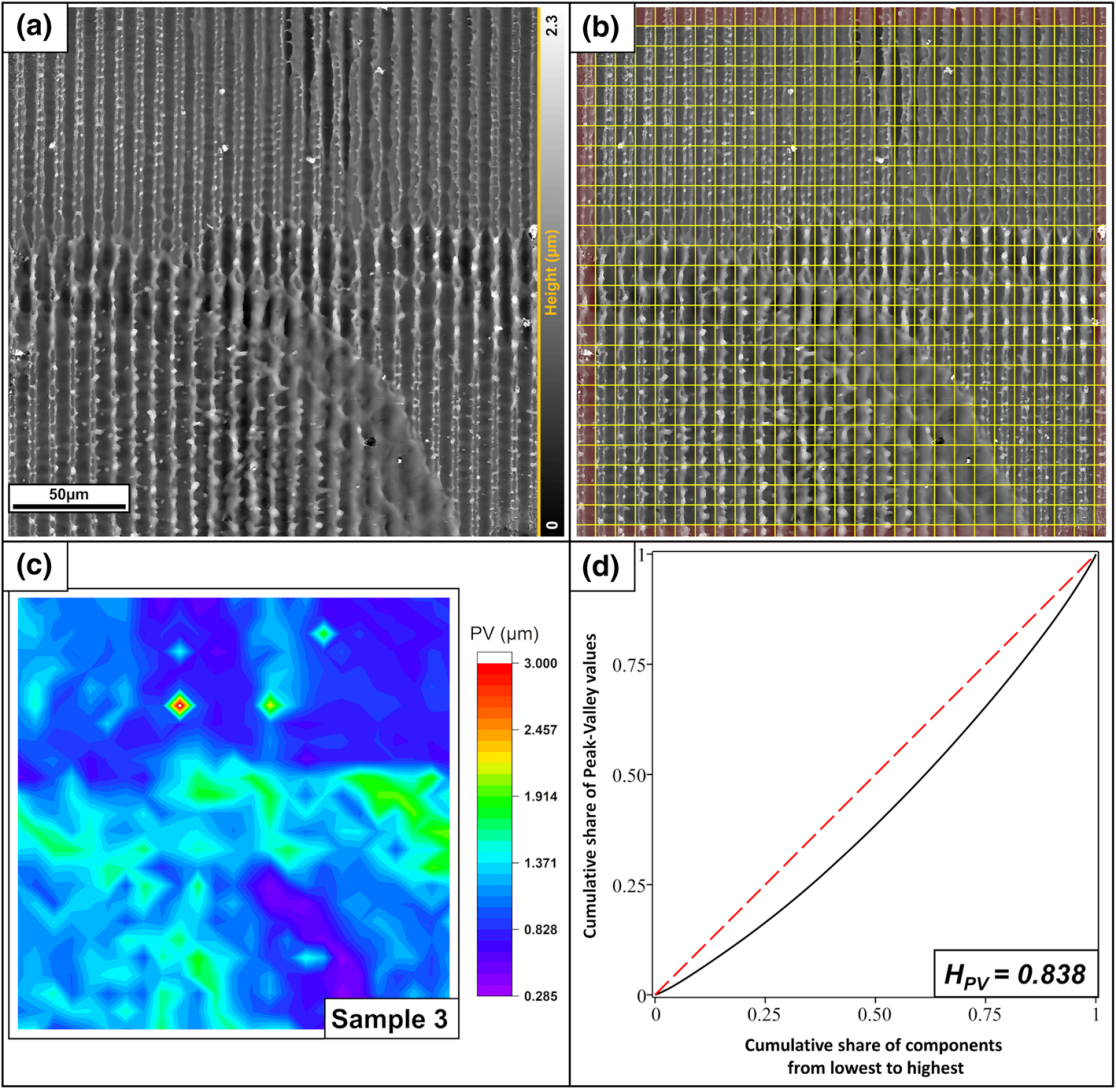
(**a**) LSM grayscale height profile image of a polished steel sample patterned with a nanosecond laser. The figure depicts the area where 4 different spots are stitched together. (**b**) Splitting into the individual components. The red area was not used to calculate the homogeneity. (**c**) Color map of the PV values. (**d**) Gini curve of the PV distances, yielding a homogeneity of H_PV_ = 0.838.

The surface depicted in Fig. 12 exhibits the overlap area of four neighboring interference spots on the steel surface produced by the nanosecond laser. The size of the periodic unit is 8.8 µm. The inner 25 × 25 cells were used for the calculation. The color map is clearly divided into different zones formed by the four spots. Owing to multiple instances of melting, some areas are flattened; in other areas, the topography maxima are not closed and there are double peaks. In addition, the melting drops are present again. As a result, the PV distance homogeneity is lower than that for the first two samples, H_PV_ = 0.838.

The calculated homogeneity values exhibit a consistent pattern: the larger the deviations of the PV distance, the smaller the homogeneity determined for the studied attribute. However, it should not be forgotten that the PV value is the difference between the highest and lowest points of a cell, i.e. the manner in which the material is deformed has no relevance.

The next step is to attempt to illustrate the difference in the shape of the material more clearly. For this, homogeneity analyses are performed once for the Mid-Height (MH) and once for the Mid-Depth (MD). In addition, they demonstrate the flexibility of the described method by selecting a non-standard parameter for evaluation.

**Figure 13:**
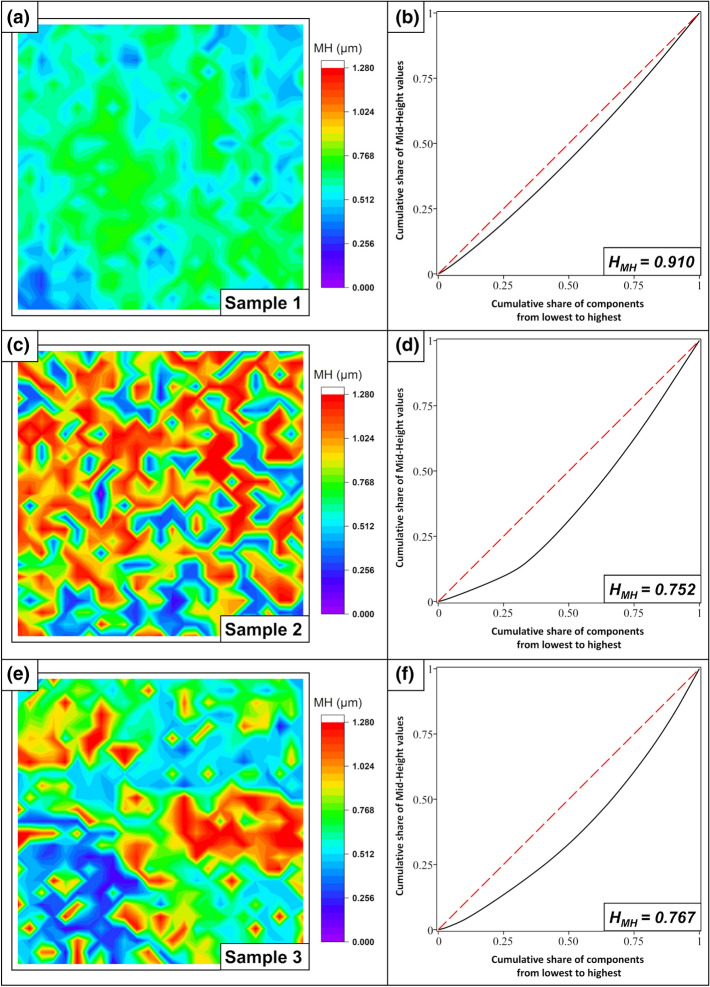
Mid-Height—Color map and Gini curve for sample 1/tungsten (**a**,**b**), sample 2/steel (**c**,**d**), and sample 3/steel with overlaps (**e**,**f**).

First, the Mid-Height for all samples is examined, as in Fig. 13:

Sample 1: Tungsten exhibits a very high homogeneity H_MH_ = 0.910 with regard to the Mid-Height attribute; the deviations are primarily explained by the rough oxidized surface. A closer examination of the oxide layer for this type of sample can be found in^34^.

Sample 2: The MH values possess a significantly higher variance than the PV values before. A closer look at the LSM grayscale height profile images (in Figs. 7 and 11) indicates that the lines of the topography maxima are not continuous, but rather show cavities, bumps, and melting droplets. This has negligible influence on the PV value but induces a strong reduction in the homogeneity with respect to the Mid-Height, H_MH_ = 0.752.

Sample 3: The textured steel surface with overlapping zones shows local areas with slightly homogeneous sections in the individual quadrants. There are areas with constant open or constant closed maxima, as well as completely melted areas. However, over the entire sample, the value varies greatly, yielding a homogeneity value of H_MH_ = 0.767.

**Figure 14:**
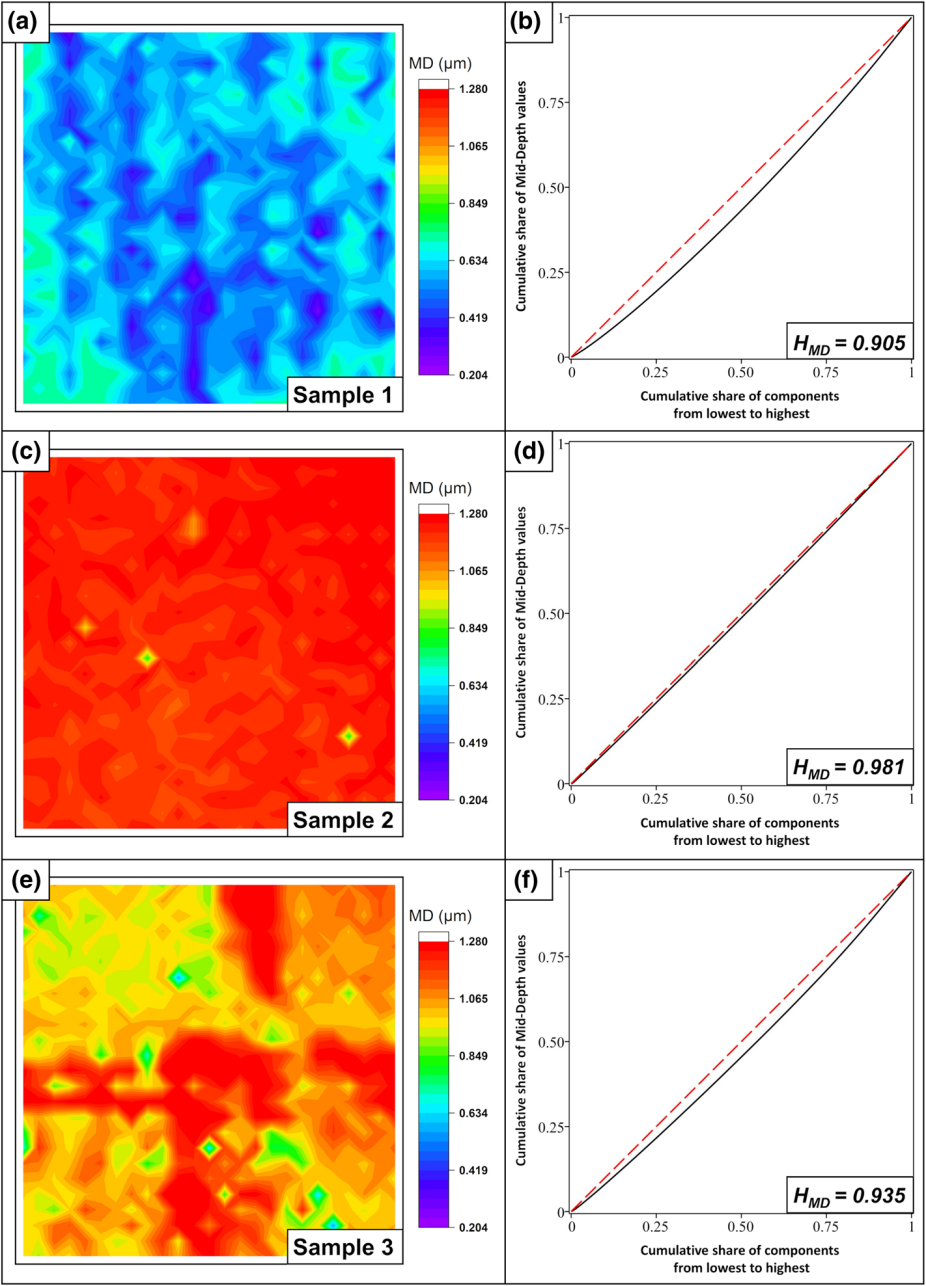
Mid-Depth—Color map and Gini curve for sample 1/tungsten (**a**,**b**), sample 2/steel (**c**,**d**), and sample 3/steel with overlaps (**e**,**f**).

Finally, the Mid-Depth for all samples is analyzed, as shown in Fig. 14:

Sample 1: Appears relatively homogeneous for the Mid-Depth H = 0.905 but is slightly worse than for the Mid-Height. The sample (Fig. 10) indicates that the minima, in contrast to the maxima, contain a slightly uneven wavy pattern. This is caused by the overlapping of the area with the laser beam when scanning the sample.

Sample 2: Appears with the exception of the melting drops as almost perfectly homogeneous H = 0.981. The explanation can be found in the manufacturing process. The energy input into the material by the nanosecond laser induces melting and smoothing of the topographic minima.

Sample 3: Here, the value is still high for the same reason as in example 2. As already explained, it comes to a melting and smoothing by the nanosecond laser. However, the value is somewhat lower, because the overlap leads to disturbance areas, H = 0.936.

From a physical perspective, the observed differences are easy to explain. In the case of the nanosecond laser, the surface is locally melted at the intensity maxima^35^. Owing to the Marangoni effect^24^, the melted areas converge towards the colder low intensity regions to produce the structural maxima on the original surface^36^. The topographic minima are simultaneously smoothed during this process. If the power of the laser is insufficient, the melting fronts do not touch each other, and cavities remain in the ridges of the topography maxima (see Fig. 7f).

If the sample surface is not flat, for example because the surface has is already structured, or if there are disturbances in the beam source, additional uneven melting occurs. This has a further influence on the shape as well as the height of the peaks and depth of the valleys and can be a major source of inhomogeneities.

The effects on the attributes can be seen in Fig. 9a, which shows a line-profile of a real surface. The first peak [I] shows a closed maximum, the others [II],[III], where the power of the laser was not sufficient, an open maximum. While the “Peak-Valley distance” is only slightly different, there are big differences in the “Mid Height”. Due to the smoothing effect, the “Mid-Depth” remains mostly constant. This explains why for the samples processed with the nanosecond laser, MD is the most homogeneous followed by PV and then MH.

In the case of the picosecond-laser, the structuring is dominated by ablation^35^. Without significant metal flow, imperfections in the topographic maxima are less pronounced, so there are no double peaks of the maxima. Which is reflected by the high level of homogeneity for PV and MH. Only the oxide formation and the overlap areas lead to a slight deterioration, which slightly affects the MD value.

Finally, the overall homogeneity of the system with respect to the selected attributes can be calculated according to formula (10), which yields the following values:

- **H**_**total-1**_** = 0.764** for the tungsten sample.
- **H**_**total-2**_** = 0.667** for the steel sample.
- **H**_**total-2**_** = 0.601** for the steel sample with overlaps

From this, it can be concluded that the picosecond laser on tungsten has created the most homogeneous structures with respect to the examined attributes "Peak-Valley distance", "Mid-Height" and "Mid-Depth". Behind it lies the undisturbed interference structure generated by the nanosecond laser and finally the surface containing the overlapping zones of the nanosecond structures.

In summary, it can be confirmed that the analysis provides reasonable results both individually and overall. Nevertheless, there are also some areas in which the method requires further investigation, which will be addressed briefly below.

### Method limitations

Although the method presented is very flexible and reliably evaluates surface qualities, there are limitations and room for improvement. First, there are restrictions in the detection of the surface periodicity. The discrete Fourier analysis is based on using rectangular pixels with finite sizes. Consequently, the calculated period and pattern orientation do not always perfectly match reality. However, these errors can be minimized to a negligible level. For this purpose, the sample should already be aligned with the horizontal or vertical axis during scanning. This reduces the error of the calculated orientation.

Second, the Gini analysis has some restrictions. No attributes with negative values are permitted for the calculation of the Gini coefficient. This would draw the Gini curve below the X-axis (compare Fig. 1) and induce a distortion of the result. There are approaches for a new definition of the Gini coefficient, as explained in^37^. However, these are still experimental and are therefore not used here, but they could be a possible option for future usage. Alternatively, another uneven distribution measure can be considered, which is an advantage of the modular method.

Finally, it is important to realize that the absolute homogeneity value is not very significant. The value is only used for comparing systems and depends strongly on the number of components and the type of attributes.

Thus, an increase in the number of components leads to a decrease in the influence of each individual component on the Gini coefficient. If, for example, the system comprises 10 components and one value deviates greatly, the error impacts 10% of the considered system. If the system is broken down into 1,000 components, the error only impacts 1 per mille of the system. The homogeneity value will therefore be closer to one than in the first case.

The same applies to the attributes. If the values fluctuate in the per mille range, it naturally has less influence on the homogeneity value than if the measured attributes differ significantly. This could be improved by appropriate normalization and the specification of a certain number of components but requires further investigation.

Nevertheless, the tendency is always correctly displayed, and only the degree of deviation will vary. This must be accounted for when interpreting the data. Additionally, this underlines that measurements of different systems can only be compared if they are performed under the same conditions with the same number of components.

Despite these restrictions, the method already shows great potential and provides meaningful results in application examples.

## Conclusion

A method was proposed to measure the homogeneity of a periodic surface objectively and reproducibly with respect to many attributes. It has been demonstrated for the first time that the Gini coefficient, which is primarily used in economics, can be applied to evaluate the homogeneity of periodic surfaces. The biggest challenge of this method is the selection of components that can be solved via Fourier analysis. Obtained homogeneity values of different samples can only be compared if the underlying data was acquired under the same experimental conditions. Taking this into account, the proposed method is a powerful tool to objectively compare different periodic surface texturing methods, either regarding a desired attribute, or to determine optimal process parameters. The described technique is extremely flexible and can be applied to almost any attribute, including self-defined properties.

It should also be emphasized that this technique does not represent a universal solution for all situations, because the Gini coefficient is only one inequality measure of many. By determining other measures along with the Gini coefficient, the limitations of the Gini coefficient, such as its restriction to attributes with positive values, can be eliminated. For example, the Theil index can be utilized to decompose the surface and account for negative values by considering the coefficient of variation. However, these methods have their own limitations, and it is important to apply suitable measures for different properties. Further research is required to achieve this. Ultimately, the method presented here is a promising tool for the analysis of periodic surfaces and could further benefit from continued investigations.

## Data Availability

The datasets generated and analysed during the current study are available from the corresponding author on reasonable request.
